# Burden and Factors Associated with Depression and Anxiety Disorders in Pediatric Asthma: A Cross-Sectional Study

**DOI:** 10.1055/s-0045-1814096

**Published:** 2025-12-29

**Authors:** Sagad O. O. Mohamed, Mohammed M. I. Ahmed, Israa Elawad

**Affiliations:** 1 Department of Pediatrics and Child Health, University of Khartoum, Khartoum, Sudan; 2 Department of Pediatrics and Child Health, Sidra Medicine, Doha, Qatar

**Keywords:** child health, asthma, anxiety, depression

## Abstract

**Objectives:**

Asthma is a prevalent chronic condition in children, often associated with significant psychosocial comorbidities. This study aimed to estimate the prevalence and predictors of depression and anxiety among asthmatic children.

**Methods:**

This cross-sectional study used data from the 2023 National Survey of Children's Health, involving 55,162 completed surveys representing a cohort of 133,963 U.S. children. Multivariable logistic regression was used to assess predictors of depression and anxiety among asthmatic children.

**Results:**

Asthmatic children had higher rates of depression (9.4 vs. 3.2% in nonasthmatics, *p* < 0.001) and anxiety (21.2 vs. 8.4%, *p* < 0.001). Significant predictors of depression and anxiety among asthmatic children included male sex, obesity, lower physical activity, poor general health status, frequent hospital emergency room visits, presence of allergies, and presence of comorbid autoimmune diseases.

**Conclusion:**

Asthma is associated with a higher psychosocial burden. The identified factors, particularly poorer health status and frequent health care utilization, highlight the vulnerable subgroups and emphasize the need for integrated care approaches, including mental health screening and support for asthmatic children.

## Introduction

Asthma is one of the most common chronic diseases in childhood globally, affecting millions worldwide and significantly impacting their quality of life. Ashma imposes a significant burden on children, leading to frequent health care utilization, missed school days, limitations in physical activity, and reduced quality of life. 1 2 3

Although the physical symptoms of asthma, such as wheezing, shortness of breath, and chest tightness, are well documented, the psychological burden associated with the condition, such as depression and anxiety disorders, remains an area of growing concern. 3 4 There is a well-established association between chronic physical illnesses and an increased risk of mental health problems. The persistent nature of asthma, with its unpredictable exacerbations, need for daily management, and potential for life-threatening episodes, can create chronic stress, fear, and feelings of being different, all of which are risk factors for depression and anxiety. 5

Untreated depression and anxiety in children with asthma can have a bidirectional negative impact. Mental health issues can lead to poorer asthma self-management, reduced adherence to medication, increased perception of symptoms, more frequent asthma exacerbations, and consequently, higher health care costs and lower quality of life. Conversely, poorly controlled asthma can exacerbate mental health symptoms. 5 6 7

The link between asthma and mental health has been investigated in only a few previous studies. However, comprehensive estimates of the prevalence of depression and anxiety among children with asthma, as well as the identification of key risk factors, are still limited. 8 Moreover, it is important to have a large, nationally representative data for better estimation of the burden and predictors of depression and anxiety disorders in children with asthma. Such findings could help health care providers in recognizing and addressing the psychosocial needs of their young patients with asthma. This study aimed to estimate the burden of depression and anxiety among children with asthma and identify predictors of depression and anxiety among asthmatic children.

## Methods

### Study Population and Data Source

This cross-sectional study used the latest available data from the National Survey of Children's Health (NSCH). The NSCH provides a broad range of information about children's health across the United States. The 2023 NSCH began in June 2023 and continued until January 2024. A total of 55,162 surveys were completed nationally by parents/caregivers of children and youth, including a cohort of 133,963 participants. The NSCH examines the physical and emotional health of children, with special emphasis on factors related to the well-being of children in the United States. The NSCH employed a complex multistage sampling design to ensure representativeness. More information about the survey is available at https://nschdata.org/

### Study Variables

The variables included in this study were selected based on the availability of the data collected. Asthma was determined using respondent reports in the NSCH survey on whether a doctor or health professional ever told the respondent that the child has asthma, and whether the child still has asthma currently. For comparison and burden estimation, children without current asthma were included in analyses assessing prevalence, but were excluded from the subsequent risk factor analysis. Depression and anxiety were determined using respondent reports in the NSCH survey on whether a doctor or health professional ever told the respondent that the child has depression/anxiety, and whether the child still has depression/anxiety currently.

We considered a broad set of potential predictors based on the available data in the survey. These variables included both sociodemographic characteristics and clinical factors. The sociodemographic variables included age, sex, family structure, income (hard to cover basics such as food or housing), physical activity (exercise or play sport for 60 minutes during last week), and health insurance coverage. The clinical factors included body mass index, child's general health status, hospital emergency room (ER) visits during last 12 months, hospital admission during last 12 months, presence of allergies, and presence of comorbid autoimmune diseases (defined by the survey as having type 1 diabetes, celiac disease, or juvenile idiopathic arthritis).

### Statistical Analysis

Descriptive statistics were used to describe data in frequencies and tables. The burden of depression and anxiety among children was estimated using prevalence proportions. Group comparisons were conducted using chi-square tests to assess the association between asthma and the outcomes (depression and anxiety). A set of candidate predictors was evaluated using multivariable logistic regression analysis. The results of logistic regression were reported as adjusted odds ratios (AORs) with 95% confidence intervals (CIs). The significance level for all analyses was set at *p* < 0.05. Survey weights were applied to account for the complex survey design, and all analyses were conducted using SPSS version 20 (SPSS Inc., Chicago, Illinois, United States).

## Results

### Baseline Characteristics

The participants' average age was 8.21 *±* 5.05 years, with 51.2% of the participants were male. The overall prevalence rate of asthma among all children was 6.6%. Among asthmatic children, 87.2% of cases were described as mild, while 12.8% of cases were described as moderate to severe. Comparative analysis based on the asthma status showed that asthma was slightly more common in males and younger age groups. Asthmatic children had poorer general health status, with only 32.2% rated as “excellent” compared with 67.4% of nonasthmatics ( *p* < 0.001). The presence of allergies was markedly higher in asthmatic children (65.0 vs. 19.1%; *p* < 0.001), as was the presence of autoimmune diseases (2.8 vs. 1.0%; *p* < 0.001) ( Supplementary Table S1 ).

- *Depression and anxiety among asthmatic children* : The overall prevalence rate of depression and anxiety among all participants was 3.6 and 9.2%, respectively. Prevalence of depression was higher among the asthmatic children (9.4%) compared with the nonasthmatic children (3.2%). Similarly, the prevalence of anxiety was higher among the asthmatic children (21.2%) compared with nonasthmatic children (8.4%). The analysis showed that asthma had a significant association with both depression ( *p* < 0.001) and anxiety ( *p* < 0.001) ( Table 1 ). The rate of depression was significantly higher among those with moderate-to-severe asthma (19.9%) compared with children with mild asthma (11.3%) ( *p* < 0.001). On the other hand, the prevalence of anxiety did not show a statistically significant difference between children with moderate-to-severe asthma (26.5%) and those with mild asthma (26.7%) ( *p* = 0.091) ( Table 2 ).
- *Factors associated with depression and anxiety among asthmatic children* : Regarding depression, the analysis showed that male sex, obesity, low physical activity, poor general health status, frequent hospital ER visits, presence of allergies, and presence of comorbid autoimmune diseases were significant predictors of depression and anxiety among asthmatic children ( Tables 1 and 3 ). The full multivariable logistic regression results for all predictors of depression and anxiety among asthmatic children are presented in Supplementary Table S2 .

**Table 1:** Regression results (AOR, 95% CI) for predictors of anxiety among asthmatic children

Variable	Comparison (vs. reference)	Anxiety	*p* -Value
Age group	>12 y vs. <5 y	1.15 (1.14–1.16)	<0.001
Sex	Male vs. female	1.01 (1.01–1.02)	<0.001
BMI	>95th percentile vs. <5th percentile	1.12 (1.11–1.13)	<0.001
Family structure	≥5 children vs. <5 children	1.44 (1.43–1.44)	<0.001
Difficulty covering basics	Very often vs. never	1.44 (1.42–1.46)	<0.001
Physical activity (60 min/wk)	Daily vs. none	0.37 (0.37–0.38)	<0.001
General health status	Below average vs. excellent	3.62 (3.58–3.66)	<0.001
Presence of allergies	Yes vs. no	1.29 (1.28–1.29)	<0.001
Autoimmune disease	Yes vs. no	1.07 (1.05–1.08)	<0.001
ER visits (12 mo)	>3 times vs. none	3.71 (3.62–3.81)	<0.001
Hospital admission (12 mo)	Yes vs. no	1.32 (1.30–1.34)	<0.001

Abbreviations: AOR, adjusted odds ratio; CI, confidence interval; ER, emergency room.

**Table 2:** Association between asthma status and outcomes (depression and anxiety)

Variable	Category	Depression	*p* -Value	Anxiety	*p* -value
Asthma diagnosis	Yes	9.4%	<0.001	21.2%	<0.001
	No	3.2%		8.4%	
Asthma severity	Mild	11.3%	<0.001	26.7%	0.091
	Moderate to severe	19.9%		26.5%	

**Table 3:** Regression results (AOR, 95% CI) for predictors of depression among asthmatic children

Variable	Comparison (vs. reference)	Depression	*p* -Value
Age group	>12 y vs. <5 y	0.80 (0.80–0.81)	<0.001
Sex	Male vs. female	1.40 (1.39–1.41)	<0.001
BMI	>95th percentile vs. <5th percentile	1.968 (1.935–2.001)	<0.001
Family structure	≥5 children vs. <5 children	1.42 (1.41–1.44)	<0.001
Difficulty covering basics	Very often vs. never	3.92 (3.86–3.98)	<0.001
Physical activity (60 min/wk)	Daily vs. none	0.32 (0.31–0.32)	<0.001
General health status	Below average vs. excellent	3.22 (3.18–3.26)	<0.001
Presence of allergies	Yes vs. no	1.23 (1.22–1.24)	<0.001
Autoimmune disease	Yes vs. no	1.32 (1.30–1.34)	<0.001
ER visits (12 mo)	>3 times vs. none	5.31 (5.16–5.46)	<0.001
Hospital admission (12 mo)	Yes vs. no	1.33 (1.31–1.35)	<0.001

Abbreviations: AOR, adjusted odds ratio; CI, confidence interval; ER, emergency room.

## Discussion

The study identified a high prevalence of depression and anxiety disorders among children with asthma. The provided results encourage the integration of mental health screening and support into routine asthma management in children, which promotes a more inclusive and effective approach to care. 9 The identified substantial burden of mental health problems represents a challenge for children, families, and the health care system. Beyond confirming the correlation between asthma and psychological comorbidities, this study adds new evidence by identifying specific socioeconomic and clinical correlates of depression and anxiety within a large, nationally representative data.

The high prevalence rates were also reported by other national and international studies on pediatric asthma and mental health. Bardach et al observed similar trends in the United States, reporting that 11.2% of asthmatic children had anxiety and 5.8% had depression, significantly higher than in children without asthma. 10 Likewise, a meta-analysis of eight studies found that the aggregate prevalence of depressive and anxiety symptoms was significantly higher among adolescents with asthma than that of healthy controls, and the risk of developing depression and anxiety was significantly higher among adolescents with asthma when compared with controls. 6

As shown in this study regarding depression, the analysis showed that male sex was a significant predictor of depression. However, this association did not exhibit a consistent pattern in the literature. 6 11 Disease severity and control were also shown to be associated with depression. As revealed in this study, more frequent hospital ER visits and hospital admission in the last 12 months were all significant predictors of increased odds of depression. Children with asthma, particularly those with severe cases, were more likely to exhibit emotional and behavioral problems than the healthy children, which could negatively impact disease management. 12 A study by Goodwin et al in the United Kingdom reported that internalizing disorders, including anxiety, were more common in children with poorly controlled asthma. 13 Although causality cannot be definitively established from observational data, the association remains clinically significant.

Conversely, increased physical activity levels were correlated with lower odds of depression. However, a main issue is the parental concerns regarding activity among asthmatic patients, as some parents consider asthma to be a barrier for children getting exercise. 14 As shown in this study, greater difficulty covering basics was correlated with increased odds of depression and anxiety, highlighting the impact of low socioeconomic status and family dysfunction on anxiety and depression.

With regard to coexisting allergy or other autoimmune disorder, the results showed that the presence of allergies and autoimmune diseases were associated with more anxiety and depression in comparison to the control group. Studies have shown that asthmatic children with food allergy and pollen/fur or dust mite allergy are at a higher risk of developing mental health issues compared with children without these conditions. 15 Furthermore, evidence from genome-wide association studies supports a role for immune dysregulation across many psychiatric disorders, including depression. 16

Regarding clinical implications, the results of this study highlight the importance of incorporating mental health screening into routine asthma care for children. Given the correlation found between asthma and psychological well-being, a multidisciplinary approach that includes behavioral health support is crucial in managing both the physical symptoms and emotional challenges faced by these patients. Moving forward, it would be valuable to assess the effectiveness of accessible mental health interventions, such as school-based programs and telehealth services, in supporting children with asthma.

The study has several limitations to be acknowledged. As a cross-sectional analysis, cause-and-effect relationships cannot be established. Additionally, the use of self-reported questionnaires may have introduced reporting bias, which may affect true prevalence. The very low *p* -values likely reflect the large survey sample size and should not be overinterpreted as evidence of strong effects, as potential model overfitting cannot be excluded. The study also did not fully account for cultural and social factors that may influence how emotional symptoms are expressed. Future research should include longitudinal designs and standardized diagnostic tools to better validate and expand upon these findings.

## Conclusion

Children with asthma experience a higher burden of depression and anxiety disorders. The identified sociodemographic and clinical risk factors, particularly poorer health status and frequent health care utilization, highlight the vulnerable subgroups and emphasize the need for integrated care approaches, including mental health screening and support for asthmatic children.
