# Supplementary material for: Burden and Factors Associated with Depression and Anxiety Disorders in Pediatric Asthma: A Cross-Sectional Study
Source: Avicenna J Med. 2025 Dec 29;15(4):165–9. doi: 10.1055/s-0045-1814096 (PMC12747768; doi:10.1055/s-0045-1814096)
Supplement: Supplementary file 1 — Supplementary Material [file 10-1055-s-0045-1814096-s202590.pdf]

Supplementary Table S1 Baseline sociodemographic and clinical characteristics of children by asthma status

Variable	Category	Asthmatics	Nonasthmatics	*p*-Value
Age group	< 5 y	30.80%	30.40%	<0.001
	5–12 y	45.00%	45.10%	
	> 12 y	24.20%	24.60%	
Sex	Female	47.60%	48.80%	<0.001
	Male	52.40%	51.20%	
BMI	<5th percentile	8.90%	8.50%	<0.001
	5th–85th percentile	47.30%	61.00%	
	85th–95th percentile	19.70%	15.00%	
	> 95th percentile	24.10%	15.60%	
Family structure	Less than 5 children	63.10%	73.60%	<0.001
	5 or more children	36.90%	26.40%	
Income (hard to cover basics)	Never	44.10%	57.20%	<0.001
	Rarely	34.10%	29.10%	
	Often	17.50%	10.90%	
	Very often	4.30%	2.70%	
Health insurance coverage	No	5.20%	6.30%	<0.001
	Yes	94.80%	93.70%	
Physical activity (60 min/wk)	0 d	11.70%	10.00%	<0.001
	1–3 d	40.90%	41.90%	
	4–6 d	28.50%	27.90%	
	Everyday	18.90%	20.20%	
General health status	Excellent	32.20%	67.40%	<0.001
	Average	62.10%	31.40%	
	Below average	5.70%	1.30%	
Presence of allergies	No	35.0%	80.9%	<0.001
	Yes	65.0%	19.1%	
Presence of autoimmune diseases	No	97.2%	99.0%	<0.001
	Yes	2.8%	1.0%	
Hospital ER visits (last 12 mo)	None	66.70%	82.30%	<0.001
	Once	21.40%	13.30%	
	2–3 times	10.70%	4.00%	
	>3 times	1.30%	0.40%	
Hospital admission (last 12 mo)	No	93.0%	97.0%	<0.001
	Yes	7.0%	3.0%	

Abbreviations: BMI, body mass index; ER, emergency room.

 © 2025. The Author(s).

Supplementary Table S2 Detailed regression results (AOR) for predictors of outcomes among asthmatic children

Variable	Category	Depression	*p*-Value	Anxiety	*p*-Value
Age group	< 5 y	Ref.	–	Ref.	–
	5–12 y	0.930 (0.922–0.938)	<0.001	0.968 (0.962–0.974)	<0.001
	>12 y	0.803 (0.795–0.811)	<0.001	1.148 (1.140–1.156)	<0.001
Sex	Female	Ref.	–	Ref.	–
	Male	1.402 (1.392–1.412)	<0.001	1.011 (1.006–1.016)	<0.001
BMI	<5th percentile	Ref.	–	Ref.	–
	5th–85th percentile	2.867 (2.821–2.915)	<0.001	1.581 (1.566–1.597)	<0.001
	85th–95th percentile	2.787 (2.740–2.835)	<0.001	1.070 (1.058–1.082)	<0.001
	>95th percentile	1.968 (1.935–2.001)	<0.001	1.117 (1.105–1.128)	<0.001
Family structure	Less than 5 children	Ref.	–	Ref.	–
	5 or more children	1.424 (1.414–1.435)	<0.001	1.436 (1.429–1.444)	<0.001
Income (hard to cover basics)	Never	Ref.	–	Ref.	–
	Rarely	1.867 (1.850–1.884)	<0.001	1.516 (1.507–1.525)	<0.001
	Often	1.946 (1.925–1.967)	<0.001	1.104 (1.095–1.112)	<0.001
	Very often	3.916 (3.857–3.976)	<0.001	1.442 (1.423–1.461)	<0.001
Physical activity (60 min/wk)	0 d	Ref.	–	Ref.	–
	1–3 d	0.672 (0.665–0.679)	<0.001	0.701 (0.695–0.706)	<0.001
	4–6 d	0.388 (0.383–0.393)	<0.001	0.483 (0.479–0.487)	<0.001
	Everyday	0.316 (0.312–0.321)	<0.001	0.374 (0.370–0.377)	<0.001
General health status	Excellent	Ref.	–	Ref.	–
	Average	1.964 (1.947–1.980)	<0.001	1.517 (1.508–1.527)	<0.001
	Below average	3.222 (3.181–3.264)	<0.001	3.619 (3.581–3.658)	<0.001
Presence of allergies	No	Ref.	–	Ref.	–
	Yes	1.230 (1.220–1.240)	<0.001	1.286 (1.278–1.293)	<0.001
Presence of autoimmune diseases	No	Ref.	–	Ref.	–
	Yes	1.319 (1.297–1.342)	<0.001	1.065 (1.050–1.080)	<0.001
Hospital ER visits (last 12 mo)	None	Ref.	–	Ref.	–
	Once	1.910 (1.894–1.927)	<0.001	1.469 (1.460–1.479)	<0.001
	2–3 times	1.884 (1.862–1.907)	<0.001	2.122 (2.103–2.142)	<0.001
	>3 times	5.308 (5.164–5.456)	<0.001	3.713 (3.615–3.814)	<0.001
Hospital admission (last 12 mo)	No	Ref.	–	Ref.	–
	Yes	1.332 (1.314–1.349)	<0.001	1.315 (1.303–1.337)	<0.001

Abbreviations: AOR, adjusted odds ratio; BMI, body mass index; ER, emergency room.

 © 2025. The Author(s).
